# Optimum Design of a Ceramic Tensile Creep Specimen Using a Finite Element Method

**DOI:** 10.6028/jres.102.003

**Published:** 1997

**Authors:** Z. Wang, C. K. Chiang, T.-J. Chuang

**Affiliations:** National Institute of Standards and Technology, Gaithersburg, MD 20899-0001

**Keywords:** ceramics, finite element method, optimum design, power-law creep, specimen design, stress analysis, stress relaxation

## Abstract

An optimization procedure for designing a ceramic tensile creep specimen to minimize stress concentration is carried out using a finite element method. The effect of pin loading and the specimen geometry are considered in the stress distribution calculations. A growing contact zone between the pin and the specimen has been incorporated into the problem solution scheme as the load is increased to its full value. The optimization procedures are performed for the specimen, and all design variables including pinhole location and pinhole diameter, head width, neck radius, and gauge length are determined based on a set of constraints imposed on the problem. In addition, for the purpose of assessing the possibility of delayed failure outside the gage section, power-law creep in the tensile specimen is considered in the analysis. Using a particular grade of advanced ceramics as an example, it is found that if the specimen is not designed properly, significant creep deformation and stress redistribution may occur in the head of the specimen resulting in undesirable (delayed) head failure of the specimen during the creep test.

## 1. Introduction

Because of their high temperature strength, oxidation and corrosion resistance, and superior wear performance, advanced ceramics (e.g., silicon nitride, silicon carbide, alumina, etc.) are promising materials for use in high temperature, load-bearing applications such as turbine engines, heat exchangers and waste incinerators. Since these devices are designed for an extended service life, their creep resistance and long-term reliability under service must be ascertained. Accordingly, stress and temperature dependent creep data are needed for designing a structural component for long-term usage at elevated temperatures.

In the ceramics community, flexural creep testing using three-point or four-point bending provides an easy way of collecting creep data, as this type of loading does not create problems in alignment and gripping. However, to obtain reliable creep data from these bending specimens which contain non-uniform stress fields, a proper data interpretation method must be implemented. Assuming the material follows a power-law creep characteristic, a deconvolution method is available for sorting out the asymmetric creep behavior from the bending data to yield both tensile and compressive creep data [1–7].

**Fig. 1:**
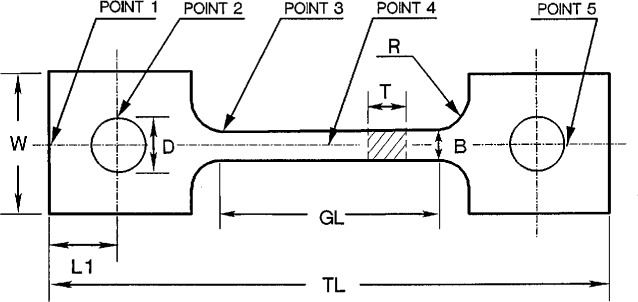
Initial design of the tensile creep specimen. Point 1: Head stress concentrator; Point 2: Hole stress concentrator; Point 3: Neck stress concentrator; Point 4: Gage section; Point 5: Load point.

In order to bypass the cumbersome task of deconvolution and data interpretation, a direct, simple tensile or compressive creep test may be adopted, even though critical issues such as alignment, fixturing, and gripping must be faced and dealt with. In the case of uniaxial tensile creep, there are a variety of testing methods that differ in terms of the design of the specimen geometry, fixturing, gripping, and the displacement measurement method (see, for example, Refs.[8–14]). All techniques have their advantages and shortcomings. We take the experimental set-up at NIST [7, 10, 15] as an example (see Fig. 1). The adopted geometry of a typical tensile creep specimen is of the coupon type consisting of two heads, a gauge section, and two ramps (or necks) connecting the two entities. A central circular hole is drilled precisely at the centerline in both heads from where the loading pins can run through to assure a good alignment. A constant load is applied at the loading pins and the separation of the two flanges attached at the gauge section is continually monitored using a laser beam for creep strain measurements [10]. In an ideal case the specimen should rupture somewhere within the gauge section at the end of the experiment or remain intact in an interrupt creep test as the nominal stress is highest, and is uniform throughout the whole gauge section. However, in many cases, the specimen breaks prematurely during the initial loading stage. Machining damage is one possible cause of the undesirable failure during fatigue testing [16], but more often premature failure is observed from the pinhole in the head or in the neck, presumably due to elastic or creep stress concentration. Since a hole or a neck is a well-known stress concentrator, a proper design of the specimen to reduce stress concentration to a minimum becomes an important task, so that the probability of rupture is enhanced in the gauge section in relation to other places (i.e., neck or head sections).

The objective of the present study was to launch an optimum design of tensile creep specimens of the type sketched in Fig. 1, so that the chances of undesirable failure can be reduced to a minimum. Owing to the complexity of the specimen geometry, finite-element analysis offers a viable vehicle to undertake the optimum design task. In this paper, we use a commercial finite element package, ANSYS,^1^ to perform the optimization analysis for the design of this tensile specimen. In order to obtain the stress distribution around the loading pinhole, contact elements were incorporated into the model for stress analysis. The creep analysis was carried out to investigate the stress redistribution and the creep deformation in high strain regions.

## 2. Original Designs

**Fig. 2:**
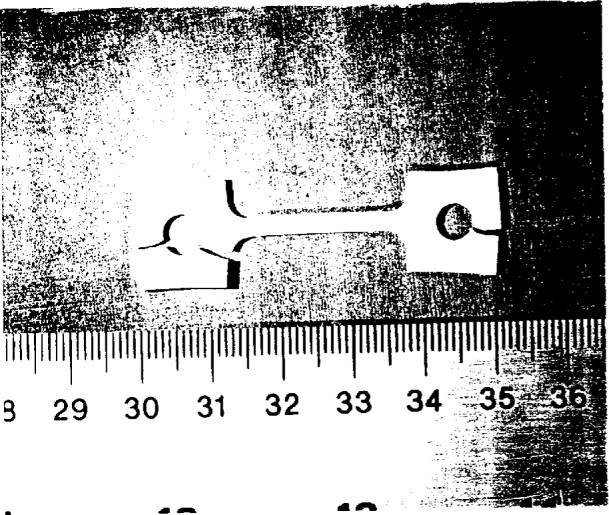
Photograph of a broken alumina tensile creep specimen after 100 h test duration.

Before we initiate the task of optimization, we briefly review the original design based on many years of experience accumulated at NIST. Figure 1 describes the original design of the tensile creep specimen geometry [7, 10, 15]. There are three different designs of specimen geometry in terms of total length *TL*; pin-hole size *D*; neck radius *R*; head width *W*; and gage length *GL*, etc. as tabulated in Table 1. For example, Specimen 1 has the following dimensions: 30.00 mm total length, 2.00 mm thickness, 10.00 mm gauge length, together with a 7.00 mm head width, 2.44 mm pinhole diameter, and 2.50 mm neck radius. A constant load is applied to the specimen from the pinhole (pin-loaded) via a shackle or clevis with a 3.05 mm opening and a pin rod of 2.44 mm diameter slide fit with a tolerance of ±0.025 mm. The whole assembly including the specimen and loading fixture is enclosed in a furnace for high temperature testing. Windows in the furnace allow the laser beam to go through for the purpose of monitoring the creep displacement. Because of the experimental set-up, the working space is somewhat limited; accordingly the total length of the specimen is set at a fixed value of 30 mm, 50 mm, or 76 mm. The current design has been used to produce tensile creep data for silicon carbide [12], silicon nitride [17], and other ceramic materials [18]. Sometimes the specimen breaks at the neck or head region at the initial loading stage. Figure 2 shows one example of such a failed sample. In order to prevent premature failure of the specimen, redesigning the specimen is clearly warranted.

From the current NIST tensile creep test program, the materials selected for study are a grade of commercially available HIPed silicon nitride with a trade name of PY-6 produced by GTE Laboratories, Waltham, MA (1989–90 vintage) Inc. for the specimen and sintered silicon carbide with a trade name of Hexolloy produced by Norton Ceramics Co. for the loading pin rod. Their mechanical properties at ambient temperature, as supplied by the vendors, are as follows:
For Si_3_N_4_:modulus of elasticity, *E* = 350 GPa Poisson’s ratio, υ = 0.24 ultimate tensile strength, *S*_u_ = 900 MPa with a Weibull modulus = 15.For SiC:modulus of elasticity, *E* = 400 GPa Poisson’s ratio, υ = 0.24 ultimate tensile strength, *S*_u_ = 500 MPa with a Weibull modulus = 17.These materials were chosen from a round robin testing program in which NIST participated.

In the present paper, we will present a two-part design analysis. In the first part, we analyze the stress and strain fields around the pin hole caused by the contact between the pin and the specimen. This will help us understand the cause of premature failure for many specimens of original design, and plan our design path in the second part of the analysis. In the second part, we systematically alter the geometry to minimize the stress/strain at the critical spots, thereby yielding the final design for the optimized geometry.

## 3. Optimum Design Procedure

### 3.1 Initiation of Optimum Design

As stated, throughout the entire design procedure, the total length of the specimen will be fixed at either 30 mm, 50 mm or 76 mm depending on the specimen class. Also, the opening of the clevis will be fixed at 3.05 mm. These two conditions are regarded as given, and are the only two constraints imposed on the problem. All other parameters will be varied to find the optimum dimensions. In the optimization scheme, we will take the current design parameters shown in Fig. 1, except the pinhole diameter, as the initial values in the iterative optimization loop. Throughout the entire creep test, since the applied load is kept constant, the nominal tensile stress in the gauge section will always be maintained at 100 MPa.

### 3.2 Design Criteria

In the optimum calculations, we set the following design criteria to be met in each iterative loop:

- Smallest stress concentration in the neck area (i.e., the transition region from the neck of the flange to the gauge section).
- Tensile stresses as high as possible in the loading pin rod, but without failures.
- Smallest critical stresses in the head.
- Longest possible gauge length.

## 4. Design Analysis

### 4.1 Pin-Specimen Contact Analysis

When we investigate the stress distribution on the specimen, particularly around the pinhole in the head, the effect of the contact surface between the pin and the pinhole must be considered because, for a given applied load, the traction forces will depend on contact area and the local stresses will be influenced by the applied traction at the pinhole. In the ANSYS software, a 2-D Point to Surface Contact Element named CONTAC48 [19] intended for general contact analysis is available. The contact element contains three nodes, one at the contact surface and the other two at the target surface, so that either a 4-node or 3-node structural element can be used to model the contacted region.

**Fig. 3:**
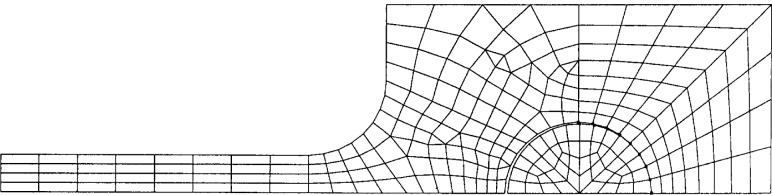
Two-dimensional finite element mesh for the quarter-specimen.

Owing to the symmetry conditions, only one quarter of the specimen geometry needs be considered in the finite element modeling. The overall 2-D finite element mesh for the quarter-specimen is shown in Fig. 3, taking the 50 mm originally designed specimen as an example. For loading conditions, we consider a point load applied at the right side of the pin as indicated in Fig. 3.

### 4.2 Creep Analysis

Since the tensile specimens are intended for use at elevated temperatures, a creep stress and strain analysis is needed to evaluate the specimen behavior during loading. The need for optimization analysis can be seen from Fig. 2 where a creep specimen failed prematurely in less than 100 h of the creep test. An undesirable failure has occurred from the pinhole, which is mainly induced by a significant creep deformation in the specimen head. Therefore we must pay more attention to the creep behavior in this region.

The finite element package ANSYS has a feature for creep calculations for most engineering materials [21] and various formulations describing creep law could be directly used in the creep analysis. Here we choose an Arrhenius type of equation in which the values of the coefficients are provided for hot-pressed silicon nitride from the literature [17].

$${\dot{\epsilon }}_{s}=A_{0}{\left(\sigma /\sigma_{0}\right)}^{n}e^{-\frac{Q}{RT}}$$ (1)

where ${\dot{\epsilon }}_{s}$ is the steady state creep strain rate, *σ*_0_ is the reference stress, *A*_0_ and *n* are materials constants, *σ* is the applied stress, *Q* is the apparent activation energy for creep, *R* is the universal gas constant, and *T* is the absolute temperature. For the purpose of computation, we consider the isothermal conditions at *T* = 1600 K in which *Q* = 1310 kJ/mol for the PY-6 material according to Krause and Wiederhorn [17]. Then, Eq.(1) reduces to the following simple power-law form:

$${\dot{\epsilon }}_{s}=A_{0}{\left(\sigma /\sigma_{0}\right)}^{n}$$ (2)

This is the well-known Norton’s equation where the material constants *A* = 5.0×10^−23^ h^−1^ and the stress exponent *n* = 8.4, if ${\dot{\varepsilon }}_{s}$ is expressed in the units of (1/h) and *σ*_0_ is 1 MPa.

In order to focus our emphasis on the pin hole area, we refine the local mesh in the maximum stress region. We choose a special type of element available in ANSYS with analyzing creep capabilities to handle the time-dependent creep strain and stress redistribution computations.

### 4.3 Steps in an Iteration

In the ANSYS software, a design optimization routine is available. The routine is capable of performing a series of analysis-evaluation-modification cycles until all specified design criteria are met [20]. The procedure of design optimization consists of the following six main steps:

#### (a) Initialize the design variables

In this paper, we performed the optimization for the tensile specimen geometry. As was shown in Fig. 1, there are four design variables: the gage length *GL*, the radius of curvature of the neck *R*, the width of the head *W*, and the location of the hole center *L*1. The initial values of these design variables, which represent the starting point in the design loop, will be later modified by the ANSYS optimizer.

#### (b) Build the model parametrically

In this step, we construct the model in terms of the design variable parameters. An 8-node structural plane element named PLANE82 [22] is used for the specimen design.

#### (c) Acquire the solution

In this step, we first define the analysis type as the *linear static analysis* and set the appropriate applied load level necessary to make the uniform tensile stress of 100 MPa in the gage section of the specimen. Then we seek the finite element solutions for each intermediate design.

#### (d) Retrieve the results parametrically and set the state variables and objective function parameters

In the case of the specimen design, there are three state variables: namely the *hole stress*, *neck stress*, and *head stress*.

#### (e) Declare optimization variables and begin the optimization process

The optimization paths for the specimen design are described in the next section.

#### (f) Review and verify the results

We review and verify the results of the optimization run by plotting the graph of state variable versus design parameter, so that we can track how a variable changed from loop to loop.

### 4.4 Optimization Paths

There are at least four independent design variables that must be determined at the end of the optimization procedure for the specimen design: head width *W*, gage length *GL*, head length *L*1, and neck radius *R*. The optimization procedure is divided into three parts with three different objective functions and dominate parameters. In the first part, designing by pinhole stress, we use *W*1 = W−*D* as an objective function dominated by maximum pinhole stress at the edge of the hole. In the second part, designing by head stress, we use *L*_H_ = *L*1−*D*/2 as an objective function dominated by head stress. In the third part, designing by neck stress, we use *L*2 = *TL*−(*GL*+*R*+*L*1) as an objective function dominated by neck stress.

## 5. Results

### 5.1 Elastic Stresses in the Specimen

Analysis results of the stress distributions for the quarter-specimen in the case of the original design for a 50 mm specimen are shown in Figs. 4a and 4b. Figure 4a is the principal stress contour plot based on contact analysis. The solution of the equivalent stress distribution is given in Figure 4b, where the equivalent stress is defined by *σ*_e_ = √ (3*σ*′*_ij_ σ*′*_ij_*/2), where *σ*′*_ij_* is the deviatoric stress tensor, and the repeated index denotes summation. As can be seen in the figures, a maximum principal stress is found on the edge of the hole which is a stress concentrator (see Fig. 4a). There are two additional stress concentration zones; one is in the neck area located at the intersection of the straight gage section and the curved neck region, and the other is located in the head at the end of the specimen along the centerline. Moreover, there exists a maximum equivalent stress at the pin and pinhole edge in Fig. 4b.

### 5.2 Pin and Pinhole Dimensions

Since it is well-known that increasing the pin diameter will result in a monotonically decreasing stress on the pin rod, it follows that the sizes of both the pin rod and the slightly larger pinhole should be as large as possible to the extent imposed by the finite physical dimension. This result is applicable to the general case of ceramic materials. For the case of a silicon carbide rod and silicon nitride specimen, the general rule of thumb is that the normalized pin diameter is approximately 0.1 (i.e., *D*/*TL* = 0.1) of the total specimen length. For example, in the case of the pin rod designed originally, *D* = 4.76 mm for the 50 mm specimens. Hereafter we will use this constraint to perform the optimization task for the 50 mm specimens, as an illustrative example.

### 5.3 Specimen Geometry

#### (a) Design by hole stress

Taking a series of hole stresses against *W*, we obtain Fig. 5a. If an allowable value of hole stress is specified for the specimen design, we can then determine the *W* value. In the same way, we get another group of values of hole stresses from Fig. 5b for a range of hole locations and determine the optimum value of hole position *L*1. By the same token, Fig. 5c is a plot of hole stress for a range of gage lengths, and the best gage length can then be determined from this plot. It turns out that the stresses are monotonically increasing for increasing gage lengths. For the sake of minimizing stress, the smallest gage length should be used. However, to fully utilize the material, the longest gage length should be adopted. Clearly a balance must be struck between these two opposing “forces.” Similarly, Fig. 5d plots the hole stresses with increasing neck radius.

#### (b) Design by head stress

For a given value of pinhole diameter *D* = 4.76 mm, Fig. 6a shows the relationship between head stress and hole position *L*1. The head length 2×*L*1 can be easily chosen from Fig. 6a, if an allowable value of the head stress is imposed for the design. Similarly, Fig. 6b plots the head stress vs head width *W* for the given *D*, and the optimum value of *W* can thus be chosen.

#### (c) Design by neck stress

**Fig. 7:**
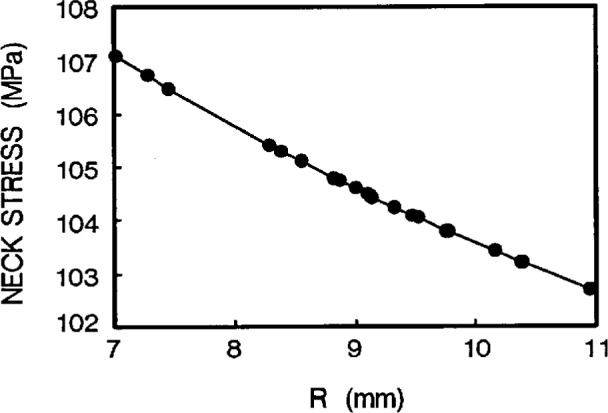
Neck stress vs neck radius *R*.

From Fig. 7, we find that the neck stress is strongly dependent upon the value of the neck radius *R*, so that if the allowable value of the neck stress is given in the design, the neck radius can be determined accordingly.

### 5.4 Allowable Stresses

A series of criteria must be given for the specimen design, including the allowable value of hole stress *σ*^c^_hole_ on the edge of the pinhole, the allowable value of head stress *σ*^c^_head_, and also the allowable value of neck stress *σ*^c^_neck_. The design criteria are expressed as follows:

$$\sigma_{\text{head}}\leq {\sigma^{c}}_{\text{head}}$$ (3)

$$\sigma_{\text{neck}}\leq {\sigma^{c}}_{\text{neck}}$$ (4)

$$\sigma_{\text{neck}}\leq {\sigma^{c}}_{\text{neck}}$$ (5)

where *σ*^c^ is the allowable stress. In the optimum design, we set the following criteria after taking the allowable stress values into account for the selected silicon nitride:

$${\sigma^{c}}_{\text{hole}}={\sigma^{c}}_{\text{neck}}={\sigma^{c}}_{\text{head}}=105\;\mathrm{MPa}.$$ (6)

We then obtain the following set of design parameters for a 50 mm total length specimen after a series of iterations:

$$\begin{array}{l} D=4.76\;\mathrm{mm}\\ W=12.5\;\mathrm{mm}\\ R=9.0\;\mathrm{mm}\\ L1=7.1\;\mathrm{mm}\\ GL=12.0\;\mathrm{mm} \end{array}$$ (7)

The same procedures have been carried out on the other specimens, i.e., a 30 mm total length specimen and a 76 mm total length specimen. The final results are given in Table 2 in nondimensional ratios which are formed via normalization to the total specimen length *TL*.

### 5.5 Creep Behavior

The stress redistribution contour for the quarter-specimen due to creep is displayed in Fig. 8. At the early stage of creep, the stress near the hole edge is high but drops dramatically with time. The stress relaxation behavior due to creep is shown in Fig. 9. At *t* = 5 h, the initially designed specimen (Fig. 9a) for example, the stress at a distance 0.27 mm from the hole is observed to have the highest stress (106 MPa), then drops to 84 MPa at *t* = 200 h. For a finally designed specimen, however, the stress level at the same location (0.27 mm) has diminished to about 80 MPa, and the highest stress (104 MPa) at the edge of the hole reduces to 76 MPa at *t* = 200 h (Fig. 9b). Overall, we see a gradual relaxation of the stress concentration around the hole for both designs of specimens with reduced stresses for the final design (see Figs. 10a and 10b).

The stress redistribution in the critical area (i.e., at the hole stress concentration zone, gage length nominal stress section, neck stress concentration zone, head stress concentration zone, and the load-point applied stress area) due to creep are displayed in Figs. 11a and 11b, respectively, for the initial and final designs of the specimen. As can be seen for both cases, relaxation takes place at the hole edge and neck root but increases slightly at the head and load-point, whereas it remains at an approximately constant level within the gage section. Again, we see that the creep stresses in the final design have been dramatically reduced. As opposed to the elastic case, the stress peak does not occur on the edge of the hole, after the stress redistribution is modified by creep. From Fig. 8, we also observe that a bigger equivalent stress (see Fig. 8b with *t*= 0 h, Fig. 8d with *t* = 50 h, and Fig. 8f with *t* = 200 h) is exactly located at the place where creep fracture may occur in the specimen. The analysis seems to suggest that creep failure follows a maximum strain criterion. Hence, if a design against early failure due to creep is to be implemented, then the stress redistribution and the total strain accumulation must be evaluated (see Figs. 10 to 12).

The creep strain solutions in the critical areas are given in Fig. 12 for both cases. As can be seen, after about 100 h the creep rates (i.e., the slopes) around the hole are almost identical. However, for the initial design, the accumulated strains at the hole edge are very high within the first 80 h (Fig. 12a). If the failure criterion of this material is such that failure will occur when the local strain exceeds the critical strain (or creep ductility), then the cracking from the pinhole will be observed at the location corresponding to this element. However, from Fig. 12b we see that the total creep strain at the hole edge has been reduced from 0.044 % to 0.015 %. Thus, based on the critical strain failure criterion, the optimized specimen should not suffer premature failure during creep test.

## 6. Discussion

### 6.1 Optimum Design Procedure

In the process of optimization, we have imposed the constraint that the total length of the specimen be fixed at either 30 mm, 50 mm, or 76 mm (see Table 1). The length of the specimen usually is dictated by the size of the billet, fixture design, and other environmental factors. Since the current optimization scheme is based on elasticity analysis, the geometry of the specimen under investigation is scalable, and nondimensional geometric parameters normalized by the total length may be used. For instance, instead of searching for the optimum head width *W*, the nondimensional width *w* = *W*/*TL* can be used. Table 2 lists the results of optimization on a group of geometric parameters. These results can be scaled-up to acquire the optimum dimensions not only for the 30 mm, 50 mm, and 76 mm specimens, but also for other specimens with different lengths.

### 6.2 Double-Reduction Design

Sometimes a situation may arise such that after the optimum design is implemented the total load applied through the pin is so high that the pin fractures first. One possible remedy is to use higher strength pins, or alternatively, the total load could be reduced. In the latter case, to reduce the total load while maintaining the same level of tensile stress in the gage section, a double-reduction design (to reduce the gage cross-section in the *z*-direction) may have to be implemented. This means that a three-dimensional finite element model must be constructed in order to perform the optimum design. A 3-D analysis is possible, but is more labor-intensive and time-consuming. We regard this as outside the scope of the present paper and thus we will not pursue this subject further.

### 6.3 Creep Asymmetry

It is known that ceramic materials usually creep less when subjected to compressive stress than tensile stress. This asymmetric creep behavior certainly applies to many grades of silicon nitrides. There are a few exceptions such as hot pressed alumina which shows symmetric behavior [23]. In the present paper, the creep properties had been assumed to be symmetrical for the ease of computations. Our results showed that for the present material being analyzed, creep symmetry seems to give rise to delayed head failure. This conclusion is consistent with the observation of the resulting delayed failure of alumina presented in Fig. 2. Moreover, no delay failure has been observed in the case of silicon nitride and silicon carbide so far which had been known to exhibit asymmetric creep behavior. Thus, it is likely that creep asymmetry may have played a beneficial role in preventing delayed head failure of the creep specimens. Proof of this contention should be an interesting task for future research.

### 6.4 Failure Mechanisms

Upon completion of the optimum design, specimens were prepared based on the dimensions obtained except the 76 mm specimen [24]^2^, and the tensile creep testing on these specimens was carried out. The results showed that the failure rate outside the gage section has been reduced significantly. In the rare cases where the undesirable failure did occur shortly after applying a load, it is always an elastic failure due to inherently existing natural flaws in the material as received, which is normal for ceramic materials. In cases where delayed failure occurs, the fracture surface showed different characteristics, indicating that creep damage starts to accumulate. The failure in this case seems to follow the critical strain criterion, as creep damage builds-up to the critical strain level.

## 7. Summary

We have performed an optimum design of a tensile creep specimen in a plate-type geometry using the commercially available finite element code ANSYS as the design tool. We set the initial conditions based on the current design being used in the NIST program. Two constraints were imposed on the optimum design calculations: (1) the total length of the specimen is set at a fixed length (say, 30 mm, 50 mm, and 76 mm); and (2) the opening of the clevis is set at 3.05 mm. Adopting an allowable stress of 105 MPa, the results of the iterative computations yield the following values regarding the final design of the specimen: *W*/*TL* = 0.25, *D*/*TL* = 0.10, *L*1/*TL* = 0.14, *R*/*TL* = 0.20, and *GL*/*TL* = 0.25. It is expected that this optimum design should give the smallest probability of premature failure as frequently encountered in the current design.

In addition, we also performed a complete time-dependent creep analysis regarding the evolution of stress and strain as functions of time-history. The results indicated that the peak stresses and stains occurred at locations different from those predicted by elastic analysis.

The predicted critical location coincides with the location where rupture actually occurred in an alumina specimen that failed prematurely before 100 h. The creep analysis seems to suggest that creep failure follows a maximum strain criterion. Comparisons of creep stresses between as-received and optimized specimens showed reduced severity, thereby lowering the probability of premature failure during creep test for the final designed specimens.

## Figures and Tables

**Fig. 4a:**
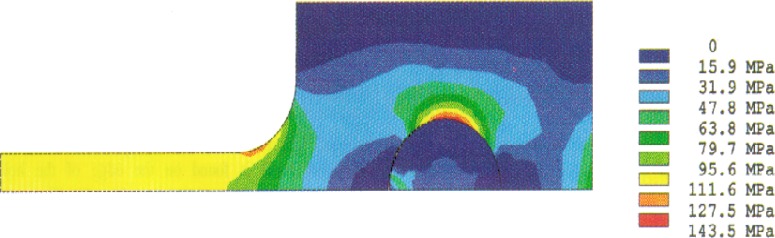
Stress distribution from the contact analysis: the first principal stress.

**Fig. 4b:**
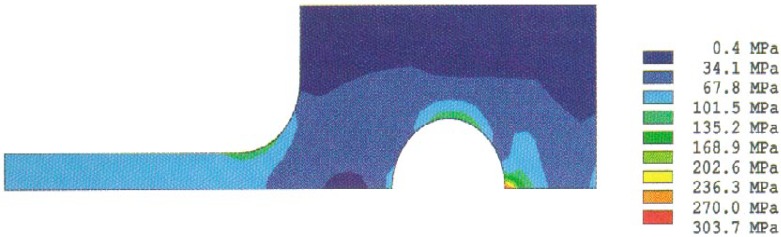
Stress distribution from the contact analysis: the equivalent stress.

**Fig. 5a:**
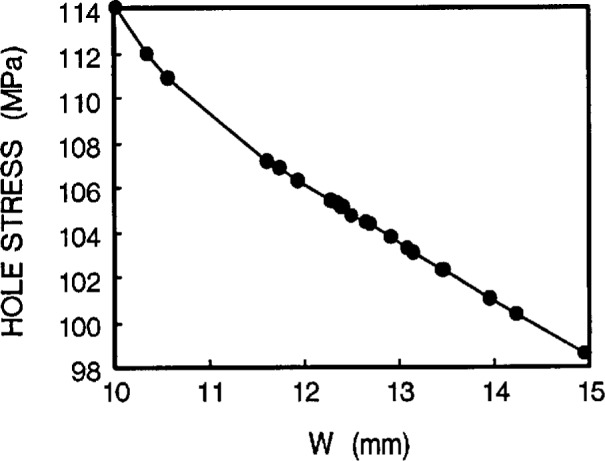
Hole stress of the specimen as a function of head width *W*.

**Fig. 5b:**
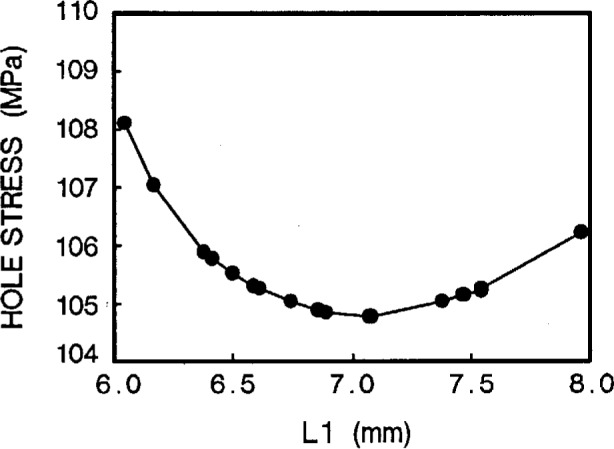
Hole stress of the specimen as a function of hole location *L*1.

**Fig. 5c:**
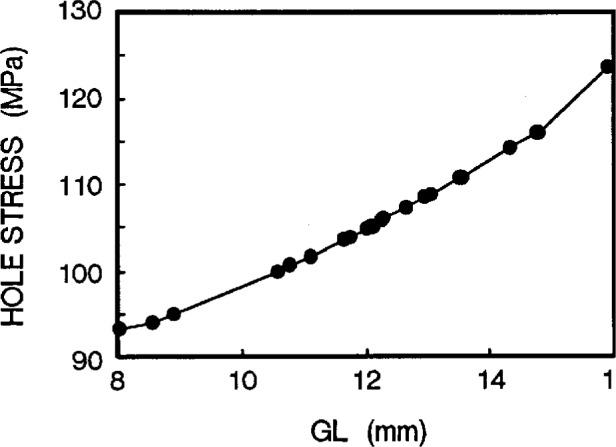
Hole stress of the specimen as a function of gage length *GL*.

**Fig. 5d:**
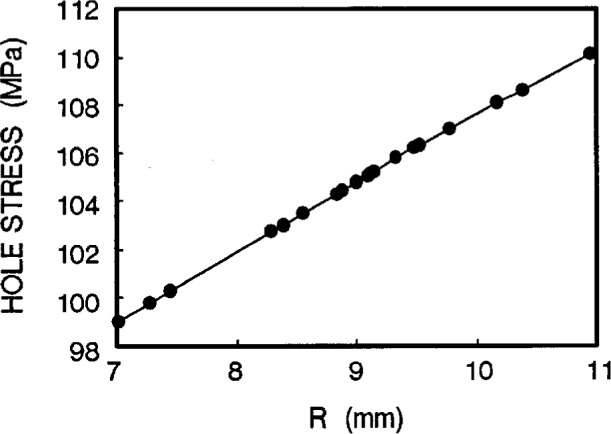
Hole stress of the specimen as a function of *neck radius R*.

**Fig. 6a:**
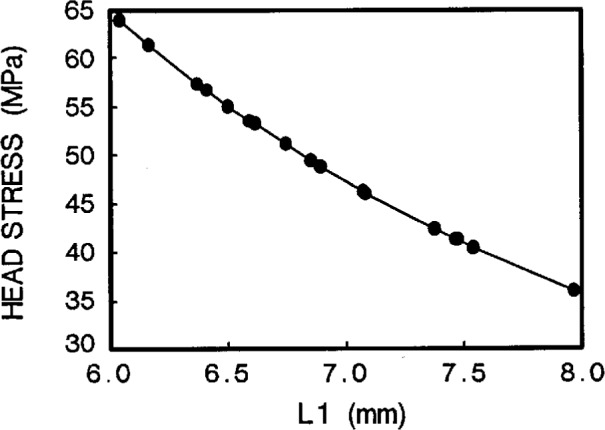
Head stress as a function of hole position *L*1.

**Fig. 6b:**
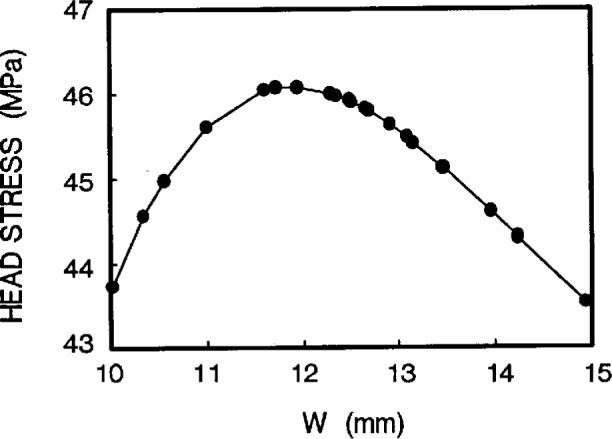
Head stress as a function of head width *W*.

**Fig. 8a:**
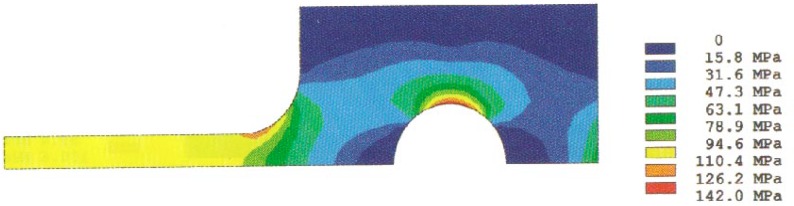
Creep stress redistribution in contour plot: principal stress at time = 0.

**Fig. 8b:**
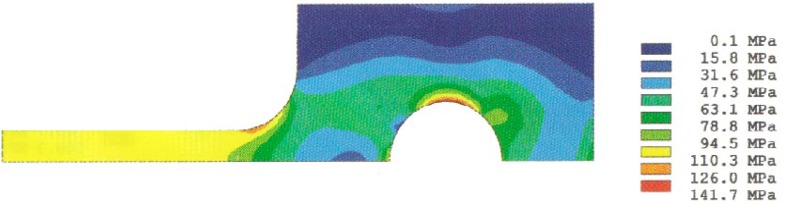
Creep stress redistribution in contour plot: equivalent stress at time = 0.

**Fig. 8c:**
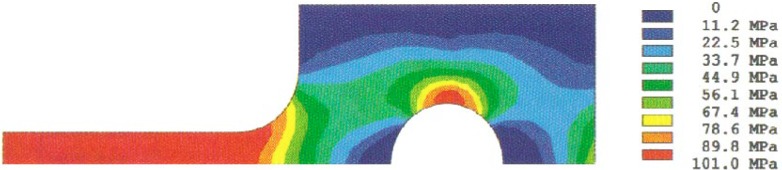
Creep stress redistribution in contour plot: principal stress at time = 50 h.

**Fig. 8d:**
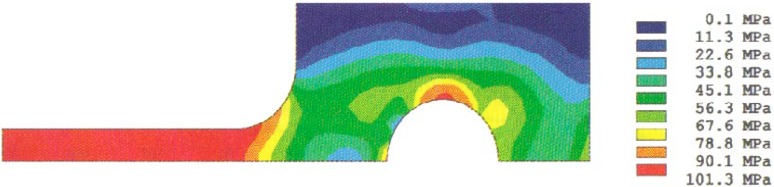
Creep stress redistribution in contour plot: equivalent stress at time = 50 h.

**Fig. 8e:**
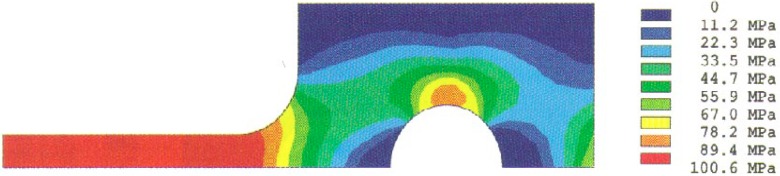
Creep stress redistribution in contour plot: principal stress at time = 200 h.

**Fig. 8f:**
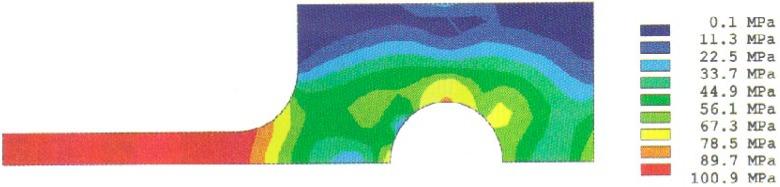
Creep stress redistribution in contour plot: equivalent stress at time = 200 h.

**Fig. 9a:**
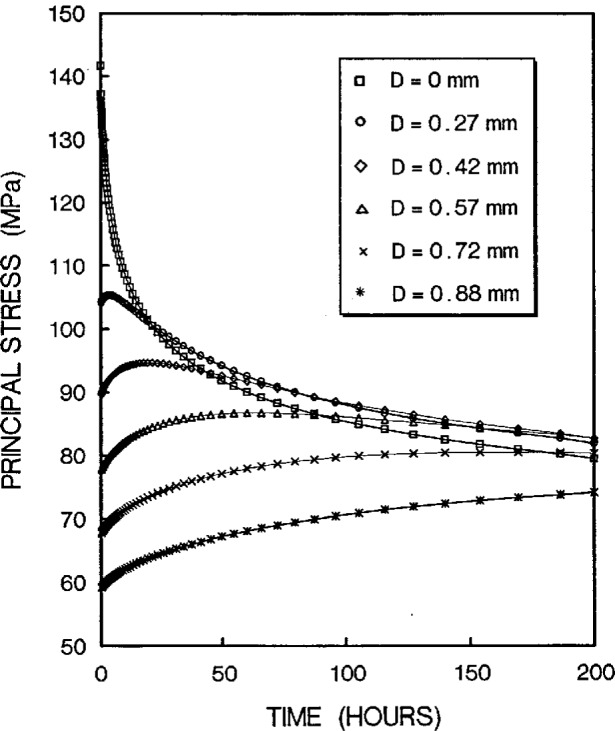
Stress relaxation at the region near pinhole as a function of time for initial specimen design.

**Fig. 9b:**
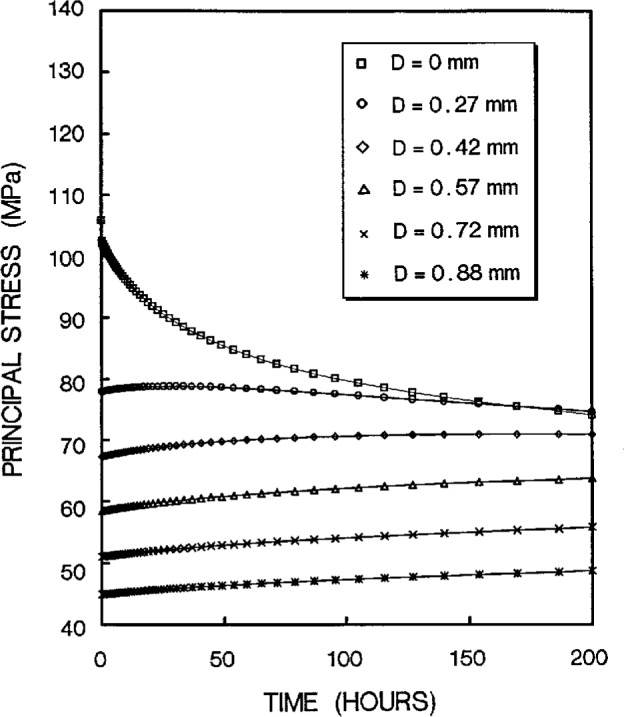
Stress relaxation at the region near pinhole as a function of time for final specimen design.

**Fig. 10a:**
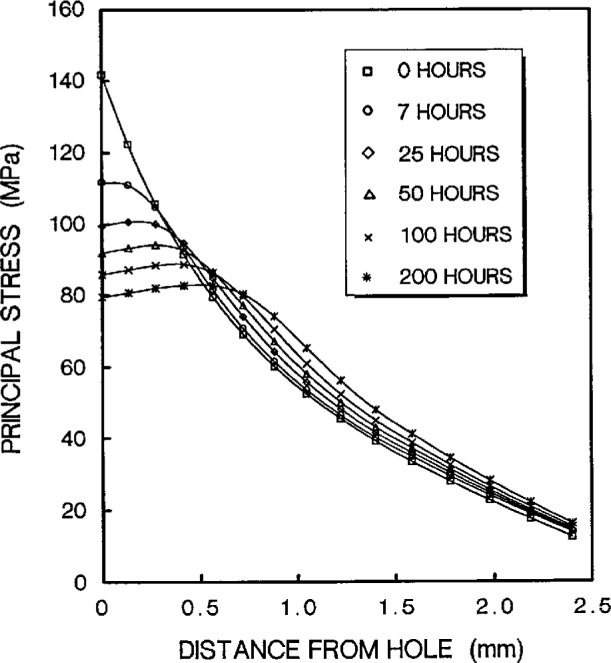
Time-dependent stress distributions in the near-hole zone for initial specimen design.

**Fig. 10b:**
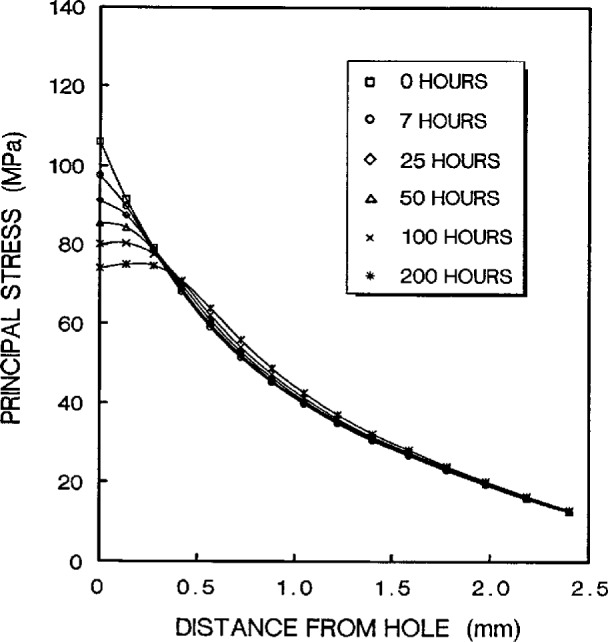
Time-dependent stress distributions in the near-hole zone for final specimen design.

**Fig. 11a:**
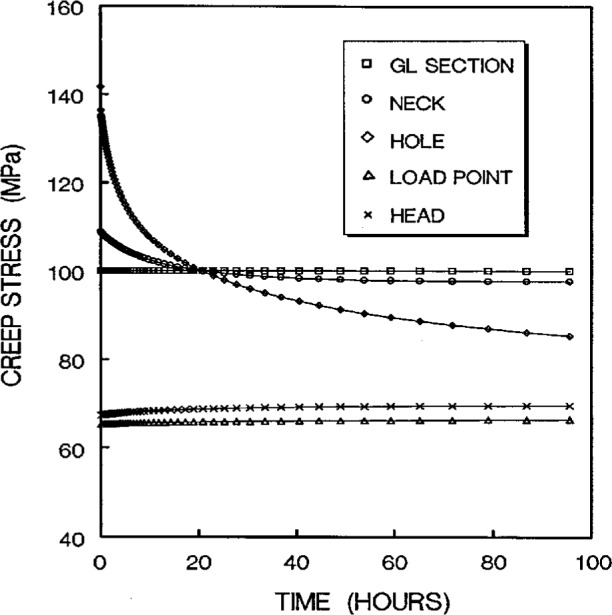
Creep stresses experienced at various critical points as a function of time for initial specimen design.

**Fig. 11b:**
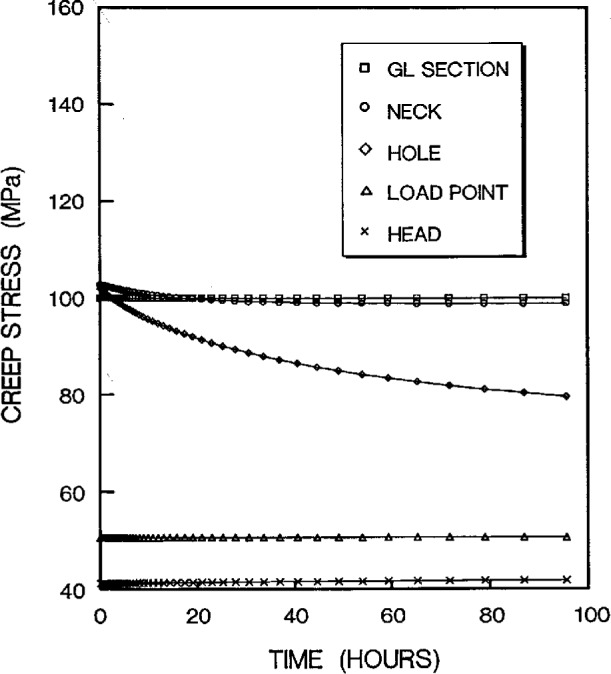
Creep stresses experienced at various critical points as a function of time for final specimen design.

**Fig. 12a:**
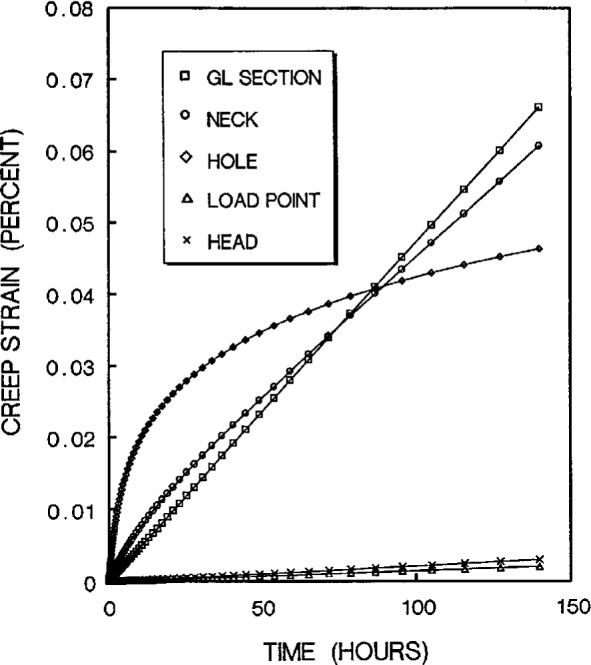
Creep strains experienced at the corresponding locations shown in Fig. 1 for initial specimen design.

**Fig. 12b:**
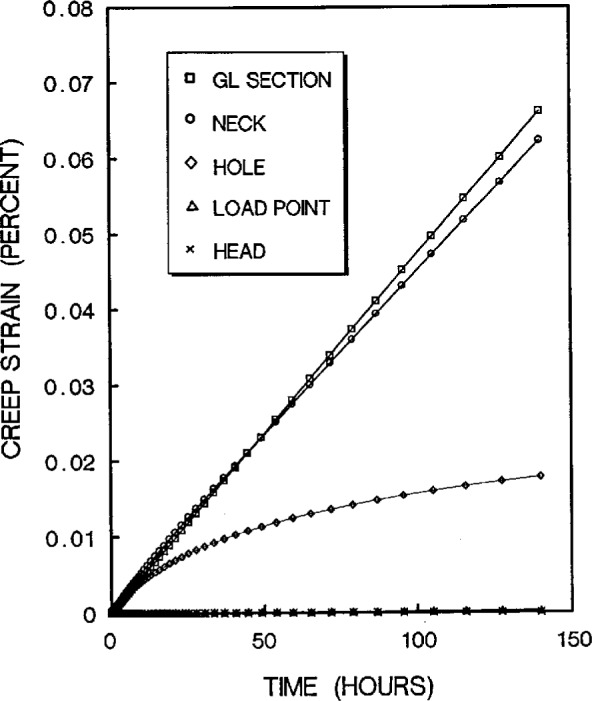
Creep strains experienced at the corresponding locations shown in Fig. 1 for final specimen design.

**Table 1:** Specimen dimensions in the initial design

Specimen No.	*TL* (mm)	*GL* (mm)	*D* (mm)	*R* (mm)	*L*1 (mm)	*W* (mm)	*GS = B*×*T* (mm)
1	30	10	2.44	2.5	4.0	7.0	2.0×2.0
2	50	20	4.76	2.5	6.25	12.5	2.5×2.0
3	76	19	4.78	19.0	8.25	15.9	2.5×2.5

Notations: *TL* = total length; *GL* = gage length; *D* = hole diameter; *R* = neck radius; *L*1 = hole position; *W* = head width; *GS* = gage section (width × thickness, *B*×*T*); see Fig 1.

**Table 2:** Sizes for final optimum design

*D*	*GL*	*R*	*W*	*L*1	*B*
0.10	0.25	0.20	0.25	0.14	0.05

a Dimension normalized to total length *TL* of a specimen.

## Footnotes

1 Certain commercial equipment, instruments, or materials are identified in this paper to foster understanding. Such identification does not imply recommendation or endorsement by the National Institute of Standards and Technology, nor does it imply that the materials or equipment identified are necessarily the best available for the purpose.

2 Since the completion of the manuscript, the dimensions of the 30 mm specimen have been altered slightly due to enlargement of the head size: *W* = 11.0 (7.5) mm; *D* = 3.57 (3.00) mm; *R* = 5 (6) mm; *L*1 = 4 (4.2) mm; *GL* = 8 (7.5) mm, where the numbers within the parentheses designate optimum dimensions.
